# Use of Doppler ultrasonography in embryo transfer programs: feasibility and field results

**DOI:** 10.21451/1984-3143-AR2018-0059

**Published:** 2018-08-17

**Authors:** Guilherme Pugliesi, Gabriela Dalmaso de Melo, Gilmar Arantes Ataíde, Carlos Augusto Gontijo Pellegrino, Júlio Barboza Silva, Cecília Constantino Rocha, Igor Garcia Motta, José Luiz Moraes Vasconcelos, Mario Binelli

**Affiliations:** 1 Departamento de Reprodução Animal, Faculdade de Medicina Veterinária e Zootecnia, Universidade de São Paulo, Pirassununga, SP, Brazil.; 2 Prole, Belo Horizonte, MG, Brazil.; 3 Embryo SYS, Ouro Fino, MG, Brazil.; 4 Departamento de Produção Animal, UNESP, Botucatu, SP, Brazil.; 5 Department of Animal Sciences, University of Florida, Gainsville, FL, USA.

**Keywords:** blood flow, corpus luteum, pregnancy. Uterus

## Abstract

The intensive use of Doppler ultrasonography in several studies in the last decade allowed the characterization of vascular perfusion and the estimation of function of the reproductive organs and tissues along the estrous cycle and pregnancy in cattle. We aim to discuss the possibility of using Doppler imaging and to explore the potential of its inclusion in reproductive programs in cattle industry. Recent studies in dairy and beef cows indicated a high accuracy and sensitivity when Doppler ultrasonography is used to evaluate corpus luteum function and to diagnosis pregnancy between days 20 and 22. Moreover, resynchronization programs starting 5 to 7 days after timed embryo transfer (FTET) coupled with early pregnancy diagnosis were developed for beef cattle, and have been implemented in commercial embryo transfer programs. These strategies allow a reduction in the interval between two FTET from ≈ 40 to 24 days and may improve the gains in reproductive efficiency when compared to traditional programs than begin resynchronization after the pregnancy diagnosis at 30 days. A second alternative to use Doppler imaging is the evaluation of luteal blood perfusion at the time of embryo transfer for selection of recipients with greater receptivity potential. This may increases fertility in FTET, as embryos would not be transferred to females with non-functional CL, and in cases with recipients surplus, females with higher receptivity would be prioritized.

## Introduction

Already in the 1980s, it was stated by the researcher Dr. O. J. Ginther that “since the introduction of transrectal palpation and the radioimmunoassay for circulating hormones, real-time ultrasonography is the most profound technological advance in the field of reproductive research and clinic of large animals” ( Ginther, 1986 ). During the last decades, the use of ultrasound imaging has reached great dimensions in research centers and commercial livestock activities, enabling great improvements in clinical diagnosis and reproductive efficiency of dairy and beef herds.

Among the several options, conventional ultrasonography in brightness mode (B, gray scale) provides a real-time, two-dimensional image of organs and structures ( Ginther, 1995 ; 1998 ). Thus, routine use of B-mode ultrasonography provides the possibility of a better evaluation of bovine females to initiate an artificial insemination (AI) program or the quality of recipients to receive embryos in embryo transfer (ET) programs. More recently, Doppler ultrasonography also started to be used in research to evaluate the reproductive system of horses and cattle throughout the estrous cycle and during pregnancy (Bollwein *e al*., 2002; Ginther, 2007 ). Its potential use is due to the ability to assess the functionality of organs and tissues based on lower or higher blood perfusion. In the last 10 years, the annual average number of research studies in the field of bovine reproduction using this technology almost doubled relative to the previous decade (14.7 vs. 7.9; ScienceDirect). Among the several possibilities for evaluation, Doppler ultrasonography has been used as a non-invasive and real-time technique to estimate the functionality of the corpus luteum (CL) for selection of recipients and for early pregnancy diagnosis in fixed-time AI (FTAI) and ET (FTET) programs (Siqueira *e al*., 2013; Pugliesi *e al*., 2014; 2016).

Thus, we aim with this manuscript to discuss the potential uses of Doppler ultrasonography in cattle and highlight recent results of its inclusion in commercial FTET programs.

## Use of ultrasonography in bovine reproduction

In applied reproductive, transrectal ultrasonography has become an important tool for evaluating the female reproductive tract in cattle. Ultrasound allowed the evaluation of reproductive organs for several purposes, such as: to monitor follicular dynamics; examination of the ovulation process; CL morphology; pregnancy diagnosis, evaluation of embryo and fetal viability; and several diagnoses of pathological changes in the female reproductive organs and tissues ( Ginther, 1995 ). Currently, its use in bovine reproductive practice focuses on the pregnancy diagnosis after 28 days, evaluation of cyclicity, and diagnosis of reproductive diseases such as ovarian cysts and uterine infections in the postpartum period ( Ginther, 1998 ).

Ultrasonography has also been constantly used in research cent and by veterinarians. However, correct evaluation and ultrasound image quality are dependent on the operator's experience and knowledge regarding the interactions between the sound wave and the tissues and organs, as well as the correct equipment setup (DesCôteaux *e al*., 2005). The greater accessibility to good quality equipment and training facilitated the incorporation of ultrasonographic evaluations into reproductive programs in dairy and beef cows. Because of these valuable features and uses, ultrasonography has become the “gold standard” for assessing the reproductive status and response of cows to reproductive programs of AI and ET ( Ginther, 1998 ; Pugliesi *e al*., 2017).

## Possible reproductive evaluations with Doppler ultrasonography

### Principles and forms of evaluation

Doppler ultrasonography is a relatively recent technique in veterinary medicine, and this equipment uses the difference between the frequencies of the reflected waves and waves sent by the transducer (“Doppler shift”; Szatmari *e al*., 2001). In blood circulation, this difference occurs due to the movement of red cells that promote a positive (higher frequency) or negative (lower frequency) difference based in the movement towards or in the opposite direction of the transducer, respectively.

**Figure 1:**
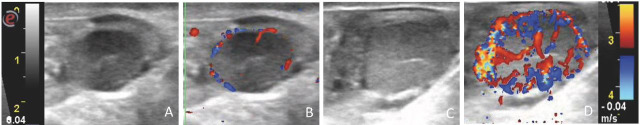
Ultrasound images of bovine ovary showing corpus luteum (CL). Panels A and B: B-mode image (A) and Color-Doppler mode (B, detection limit: 0.04m/sec) of a non-active CL; Panels C and D: B-mode image (C) and Color-Doppler mode (D-detection limit: 0.04m/sec) of an active CL.

Most of the currently Doppler ultrasound equipments allow three modes of assessing blood perfusion: spectral mode (pulse-wave), power-doppler mode and color-doppler mode. The Spectral mode allows the difference in frequency detected by the instrument to be projected on a two-dimensional chart as a function of time, and a Doppler waveform is formed during the cardiac cycle when assessing blood flow in arteries ( Ginther, 2007 ). In this mode, some indices are automatically calculated by the equipment software (resistance and pulsatility indices) and are useful for estimating blood perfusion in tissues irrigated by the assessed vessel ( Ginther, 2007 ). In Color-Doppler equipment, frequency differences are coded as colored signals over a conventional B-mode image ( Figure 1 , Panels A and B). Positive (blood flow towards to transducer) and negative (blood flow in the opposite direction of the transducer) differences are indicated by different colors, which are usually in shades of red to yellow and blue to green (Ginther *e al*., 2007). The Power-Doppler mode allows the measurement of blood flow intensity (i.e., the number of blood cells moving in the vessel per unit of time) and the image shows different color intensity according to the intensity of the flow in each point of the evaluated area ( Ginther, 2007 ). Colored representations of blood perfusion on the screen can be estimated by the proportion of tissue with colored signals or calculated by software on the amount of colored pixels ( Ginther, 2007 ; Pugliesi *e al*., 2014). Another alternative for evaluation is the subjective determination through a scale of 0 to 4 for the evaluated area ( Silva and Ginther, 2010 ; Lemes *e al*., 2017).

### Evaluation of uterine blood flow

Among all the possible applications of ultrasonography, scanning the uterus for detection of pregnancy and infections are the main commercial practices in the bovine industry. In cattle, Doppler ultrasonography was initially used to detect fetal circulation ( Mitchell, 1973 ). Also, uterus hemodynamics can be accessed by color and spectral modes, to evaluate, respectively, blood perfusion in the endometrium and mesometrium, and resistance and pulsatility indexes in the middle uterine artery (Bollwein *e al*., 2016).

Blood perfusion in the bovine uterus has a well-defined pattern during the estrous cycle. During proestrus and estrus, high values of blood flow are observed; while at diestrus, the flow remains at a low, but constant rate (Bollwein *e al*., 2016). These changes are directly associated with the circulating concentrations of progesterone (P4) and estradiol. In pregnant cows, at the beginning of the third week, there is an increase in blood perfusion in the pregnant horn compared to the non-pregnant horn ( Silva and Ginther, 2010 ). However, due to the great variability in the blood flow among animals, it is not possible to make an early pregnancy diagnosis with a simple measurement of the uterine blood flow using Doppler ultrasonography ( Silva and Ginther, 2010 ).

### Evaluation of ovarian structures

Due to the ability to indirectly assess the function of structures and tissues, Doppler ultrasonography was widely used to evaluate the function of the dominant follicle and CL throughout the estrous cycle in mares and cows (Acosta *e al*., 2005; Siddiqui *e al* ., 2009; Herzog *e al*., 2010; Ginther *e al*., 2014), and more recently, in small ruminants (Balaro *e al*., 2017). Several studies indicated a positive association between vascularization and follicular function (reviewed in Viana *e al*., 2013), since the development of follicles is related to the formation of the vascular network in the theca interna. Thereby, a higher blood perfusion is observed in the follicular wall of dominant and pre-ovulatory follicles compared to small follicles (Miyamoto *e al*., 2006).

Similarly, treatments that promote greater growth of the dominant follicle result in a more vascularized pre-ovulatory follicle with a greater capacity for estradiol secretion (Mesquita *e al*., 2014; Pugliesi *e al*., 2016a). Therefore, evaluation of the wall vascularization in dominant follicles at the onset of pro-estrus or pre-ovulatory follicles could indicate follicular function and be a tool to predict pregnancy success. However, in cattle the evaluation of the vascularization in the wall of the pre-ovulatory follicle on the day of FTAI did not indicate any relation with pregnancy rate (Pinaffi *e al*., 2015).

Ultrasonography assessment of CL with consequent estimation of its functional status is an important aspect for reproductive management and was initially developed as a diagnostic tool in addition to transrectal palpation in cattle. Although the assessment of luteal size is positively correlated with circulating P4 concentrations, during the CL regression period this correlation is lower because the rates of decrease are faster for P4 than for CL size (Kastelic *e al*., 1990; Assey *e al*., 1993). Thus, evaluation of vascularization in luteal tissue may represent more accurately the function of CL due to the high vascular network present in this transient endocrine gland (Bollwein *e al* ., 2002, 2012).

In addition, evaluation of blood perfusion in CL can be very useful because higher P4 concentrations at the beginning of the diestrus are related to the greater development of the conceptus ( Mann and Lamming, 2001 ), and associated with a higher pregnancy probability. As observed in follicles, increased luteal vascularization at early diestrus (days 4-7 after ovulation) also indicated a greater chance of pregnancy success in one study (Pugliesi *e al*., 2016a), but not in another (Pinaffi *e al*., 2015). These differences can be derived from the different moments and criteria of the evaluations carried out in these investigations.

### Luteolysis detection for early pregnancy diagnosis

Although discrete anechoic structures suggestive of the embryonic vesicle may already be observed by ultrasound between 12-14 days of pregnancy ( Pierson and Ginther, 1984 ) and the embryo can already be identified between 19-24 days (Hazen and Delsaux, 1987), ultrasound-based pregnancy diagnosis in B-mode is only recommended after 28-30 days (Pieterse *e al *., 1990). This occurs because the sensitivity and accuracy in the conceptus visualization only reach 100% when diagnosis is performed after this stage of gestation (Nation *e al*., 2003). However, in non-pregnant cows, estrus will normally return between 18 and 24 days after ovulation, once CL regression takes place, which normally occurs between days 15 and 18 of the cycle (Ginther *e al*., 2010; Pugliesi *e al* ., 2013). Thus, it has been suggested that CL evaluation at time points close to its regression or maternal recognition of pregnancy could allow an earlier assessment to gestational status (Pugliesi *e al*. 2014; Scully *e al*., 2015).

The use of ultrasonography for early pregnancy diagnosis (first 21 days of pregnancy) was initially evaluated by Kastelic *e al*. (1989, 1991). These authors reported that the assessment of CL size using B-mode provided low accuracy (<75%) before day 18 after insemination. However, higher accuracy (90-100%) was observed when the evaluation is performed between days 20 and 22. Although assessing CL size provides good accuracy, circulating P4 concentrations, which indicate luteal function, have a greater correlation with blood perfusion than with CL size during the luteolytic period in ruminants (Herzog *e al* ., 2010; Balaro *e al*., 2017; Rocha *e al*., 2017). This possible advantage allowed more in-depth studies on blood perfusion in spontaneous and induced luteolysis in cows and heifers (Ginther *e al*., 2007; 2010; Shrestha *e al*., 2011; Pugliesi *e al*., 2012). Other studies (Utt *e al*., 2009; Pugliesi *e al*., 2014; Scully *e al* ., 2014; 2015) also characterized the changes in CL vascularization between pregnant and non-pregnant cows. The results allowed the definition of vascularization characteristics and size of CL during luteolysis and served as a basis for the conception of a criterion to identify a functional or non-functional CL (Pugliesi *e al*., 2014).

Some studies indicated that Doppler ultrasonography could be used as a method for early pregnancy diagnosis (Matsui and Myamoto, 2009; Quintela *e al*., 2012). Siqueira *e al* (2013) reported a high accuracy and almost 100% of sensitivity on early diagnosis of non-pregnant dairy cattle at 20 days after insemination using just CL vascularization as a criterion. In beef herds, we observed 100% of sensitivity and 91% of accuracy when considering CL vascularization and size to determine pregnancy status on day 20 post-AI (Pugliesi *e al*., 2014). These results suggest that Doppler is an accurate tool for early pregnancy diagnosis because there is a low possibility of erroneously diagnosing a pregnant cow as non-pregnant (false negatives near to 0%).

In Siqueira *e al*. (2013) females were considered non-pregnant when they did not show colored signs indicating blood flow in the central region of CL. In Nelore beef cows (Pugliesi *e al*., 2014), it was determined that non-pregnant females would have colored signs indicating blood flow ≤25% of its area and size <2cm^ 2^ ( Fig. 1 ). This subjective evaluation can be transformed into a simpler and more practical scale through vascularization scores (Pugliesi *e al*., 2017). Thus, it is easier to understand and evaluate CL functionality.

When reduced CL blood perfusion and size are used together to identify a non-pregnant female, there are lower chances of false negatives, because the criterion becomes more rigid, avoiding the underestimation of vascularization, false negative results and economic loss (Pugliesi *e al*., 2014). On the other hand, the proportion of false positive results (proportion of cows diagnosed as pregnant by Doppler, but actually not pregnant by conventional ultrasonography on day 30) should also be considered. False positive can occur due to several factors that lead to the presence of a functional CL on the day of diagnosis, such as late ovulation to the synchronization protocol and longer estrous cycle (>22 days) in some animals. However, most of the false positives results observed with Doppler ultrasonography may come from embryo losses between early diagnosis (days 20-22) and conventional diagnosis (day 30). This is indicated by the higher proportion of false positive results in dairy cattle (Siqueira *e al*. 2013) compared to beef cattle (Pugliesi *e al*., 2014), in which pregnancy losses are normally lower (Diskin *e al*., 2016).

## Applications in commercial FTET programs

The possibility of evaluating CL functionality during the estrous cycle allowed the development of some techniques to include Doppler ultrasonography into FTAI and FTET programs. Nevertheless, ultrasound equipment should be appropriately configured for the chosen brand and model, and this is extremely important because the amount of colored signals on the display is highly influenced by the type of setting (frequency, number of frames per second, power, Doppler gain and pulse rate). In general, it is suggested a configuration that allows a minimum detectable velocity around 4-6 cm/sec ( Ginther, 2007 ). Although the cost of a portable Doppler machine is still 3 to 4 times greater than the cost of B-mode equipment, it has been reducing over the last years, which made it possible for practitioners to acquire such equipment for commercial use in FTAI and FTET programs.

### Use for early pregnancy diagnosis in FTET programs

**Figure 2:**
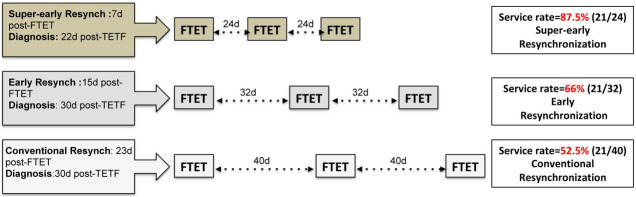
Schematic illustration of three different strategies of resynchronization in fixed time embryo transfer (FTET) programs in cattle. Super-early resynchronization may starts between 5-7 days post-FTET and a pregnancy diagnosis by Doppler ultrasonography is needed between 20-22 days of pregnancy. Early resynchronization usually starts 15 days post-FTET and conventional pregnancy diagnosis is performed on day 30 of pregnancy. Conventional resynchronization is performed after pregnancy diagnosis on day 30 of pregnancy. Service rate was calculated as the proportion of a 21-day period of normal estrous cycle in the period between two consecutives FTET procedures of each strategy.

Diagnosing non-pregnant cows with high accuracy between 20 and 22 days post-insemination allowed the development of new strategies to reduce the interval between FTAI or FTET procedures in a breeding season ( Fig. 2 ). Among these strategies, performing a second FTET in an interval of only 24 days after the first FTET is highlighted. This promotes a 16-day reduction in the interval between two FTET when compared to the traditional system, which only start resynchronization of ovulation in non-pregnant females 23 days after ET (30 days of possible pregnancy), or an 8-days anticipation compared to the early system that resynchronizes all cows at 22 days of pregnancy. However, this anticipation is only possible with the early pregnancy diagnosis by Doppler ultrasonography between 20-22 days and an early resynchronization of ovulation 5-7 days after FTET (12 to 14 days of the estrous cycle).

The possibility of initiating resynchronization after early diagnosis with Doppler was initially evaluated in 165 embryo recipients at 21 days, aiming to improve reproductive management in FTET programs (Guimarães *e al*., 2015). In this study, the early pregnancy diagnosis was performed by evaluation of CL vascularization score, in addition to the information about the presence and side of CL evaluated on the day of embryo transfer (day 7). This helped to distinguish young CLs, which have a reduced size, but high vascularization. When compared to the conventional pregnancy diagnosis on day 30, accuracy and sensitivity of early diagnosis at day 21 observed were 88.3% and 100%, respectively. This diagnosis enabled 80% of non-pregnant recipients to be diagnosed at 21 days and resynchronized for a new FTET program.

Recently, we evaluated the reproductive performance of beef recipient cows evaluated by Doppler ultrasonography to detect CL regression at day 22 of pregnancy and submitted to two protocols for FTET in 24 days (Pellegrino *e al*., 2018; unpublished data). In this study, suckling Nelore cows were submitted to a P4/estradiol based protocol for FTET. On Day 7 (Day 0 = expected ovulation), cows were evaluated by transrectal ultrasonography using a B-mode and color Doppler ultrasound instrument and received a fresh *in vitro *-produced embryo. On Day13, resynchronization was initiated by treating cows with high doses of P4 (new P4 intravaginal device and 100 mg of injectable P4), as administration of estradiol esters at this phase of the cycle may induce CL regression (Vieira *e al *., 2014). On Day 22, P4 devices were removed and cows were evaluated by ultrasonography. On Day 22, 48.3% (102 of 211) of recipients were diagnosed as non-pregnant by Doppler ultrasonography (CL with <25% of color signals indicating blood perfusion in the luteal area), ovulation was induced with 1 mg of estradiol cypionate and 25 mg of dinoprost tromethamide was given. On Day 31, non-pregnant cows were evaluated and those with a new CL received an embryo. This strategy allows a 24d-interval between FTETs. On Day 80, 67.8% (75/109) of recipients diagnosed as pregnant on Day 22 were still pregnant. At the second FTET, the utilization rate of recipients was 81.4% (83/102) and pregnancy rate for transferred recipients was 48.1% (38/79). Cumulative pregnancy rate after first and second FTET was 53.6% (113/211). These results indicated that the use of early detection of non-pregnant cows at Day 22 by Doppler ultrasonography associated with resynchronization of ovulation 6 days after FTET results in suitable pregnancy and utilization rates.

Because of the high cost of a portable Doppler equipment, the single use of CL size accessed by B-mode ultrasonography for the detection of luteolysis was recently evaluated by our group (Ataide Jr *e al*., 2018; FMVZ, Universidade de São Paulo, Pirassununga, SP, Brazil; unpublished data), but results indicated a lower accuracy and a high rate of false-negative exams. The reproductive performance of beef recipients was evaluated by B and Doppler modes ultrasonography to detect CL regression and submitted to two protocols for FTET in 24 days. Pregnancy diagnosis on Day 22 in the B-mode method was compared with the Doppler mode (gold standard). For the B-mode evaluation, cows with a CL <2cm^2^ were considered non-pregnant. Pregnancy diagnoses agreed between B and Doppler modes in 95.3% (201/211). The incorrect results were 8 false-negatives (non-pregnant for B-mode but pregnant for Doppler method) and 2 false-positives (pregnant for B-mode but non-pregnant for Doppler method). For FTAI, a similar comparison between B-mode and color-Doppler evaluation on day 22 of pregnancy indicated a higher false negative rate (15-20%). This proportion was greater in heifers, which have smaller CL compared to cows (Pugliesi *e al*., 2018; FMVZ, Universidade de São Paulo, Pirassununga, SP, Brazil; unpublished data).

Early resynchronization can shorten the breeding season, providing more days of weight gain to calves that are born earlier, especially when compared to resynchronization after the conventional pregnancy diagnosis (after 30 days of pregnancy). However, future studies are needed to define the best hormones and doses for synchronization of follicle wave emergence 5-7 days after ET, in the mid-cycle phase. First attempts indicated that estradiol esters might have a negative effect on CL maintenance, since in Holstein cows treatment with 1.5 mg of estradiol benzoate 13 days after FTAI induced luteolysis (Vieira *e al* ., 2014). Therefore, treatments with high doses of injectable P4 may efficiently suppress gonadotropins and induce new follicle wave, and hence have been preferred (Cavallieri *e al*., 2018). Nevertheless, recent data from our group have indicated that lower doses (1 mg) of estradiol benzoate or an injectable formulation of estradiol and P4 on day 14 after FTAI did not impair CL function or pregnancy maintenance in Nelore heifers (Motta *e al*., 2018; unpublished data). The choice of the resynchronization model depends on the production system adopted in each farm, and availability of the veterinarian’s staff for pregnancy diagnosis exams.

### Selection of recipients in FTET programs

In ET programs following estrus detection or fixed-time, one of the most important factors to determine the use of a recipient is the presence of a good quality CL. This quality is traditionally evaluated through the selection of recipients by transrectal palpation or by gray-scale ultrasonography to determine CL size. However, these strategies may result in ET to a non-receptive uterus due to the presence of a low or non-functional CL, since luteal functionality (P4 secretion) cannot be accessed by these evaluations. Thus, Pinaffi *e al*. (2015) evaluated recipients using Doppler ultrasonography on the day of ET and retrospectively classified these females as having Low (<40% of colored signals indicating CL vascularity) or High (>40%) luteal vascularization. Despite the small number of animals used in this study, it was verified that none (0 out of 12) of the recipients with low vascularization in CL were later diagnosed as pregnant; however, the pregnancy rate was 48.4% (n = 15/31) in recipients with high luteal vascularization. In this study, even with the difference in the proportion of vascularization between groups, the mean CL diameter was similar in cows with low (17.5 mm) or high (17 mm) CL vascularization.

**Figure 3:**
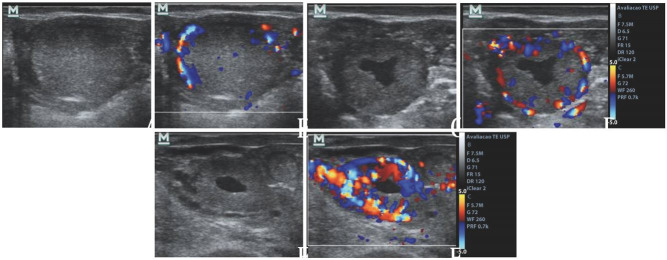
Ultrasound images of bovine ovary showing corpus luteum (CL) and follicles. Panels A, C and E: B-mode images (brightness, gray-scale); Panels B, D and F: Color-Doppler images (detection limit: 0.04m/sec) indicating, respectively, low, medium and high CL blood perfusion.

Recently, we studied (Pugliesi *e al*. 2016b) the impact of size and luteal blood perfusion assessed by Color Doppler ultrasonography at the time of ET on pregnancy rates in crossbred recipients (n = 329) with estrous cycle synchronized to allow FTET. The recipients were retrospectively divided into two subgroups according to CL size (small [<3cm^ 2^] or large [>3cm^2^]) and three subgroups according to luteal blood perfusion (low [<40%], medium [45 50%] or high [>55%]; Fig. 3 ). Cows where CL was present but was not active (<25% of blood perfusion; Fig. 1 ) were not used (9.5%). Only luteal blood perfusion affected pregnancy rate. This reflected a progressive increase in the pregnancy rate associated with increased luteal vascularization (low, 45.1%, [37/82]; medium, 55.9% [57/102]; and high, 62.3% [38/61]). In a subsequent experiment (unpublished data), recipients were evaluated on the day of FTET and the CL classified according to luteal vascularization proposed above and a classification of scores (0 to 4; 0 = no perfusion and 4 = high perfusion). Agreeing with previous results, we observed a 41% increase in pregnancy rate in recipients with high luteal vascularization compared to the ones with low vascularization. Similar difference were also observed using the central CL vascularization scores system, in which the pregnancy rate was 29% greater in cows with score 3 (62%, 44/71) compared to scores 1 and 2 (48.2%, 53/110). Although serum P4 concentrations have only differed between low and high categories, it is believed that the greater pregnancy rate in recipients with high luteal vascularity is related to the greater secretion of P4 by luteal tissue.

Thereby, it can be observed from these studies that Doppler ultrasonography can be used to discard recipients with a non-functional CL and select recipients that would have a better receptivity. This would be an alternative to increase the chances of pregnancy establishment in higher rank embryos. Also, Doppler imaging could promote a gain in reproductive efficiency in FTET programs since there would be less embryo loss, as embryos would not be transferred to females with non-functional CL, and in cases of extra recipients numbers, females with medium to high luteal vascularization would be prioritized.

## Final considerations

Color-Doppler ultrasonography is a recent and very effective tool to evaluate luteal function in recipient females submitted to ET programs. Doppler imaging can be used to identify non-pregnant recipients earlier by detecting luteolysis or to select the recipient with better receptivity at the moment of ET. The accuracy of pregnancy diagnosis is high when performed among days 20 and 22, despite the different criteria and characteristics considered by the different research groups. Thus, Color-Doppler ultrasonography is a non-invasive and real-time method to estimate luteal activity, and may be used as an innovative strategy for early diagnosis of pregnancy after FTET. In addition, evaluation of CL blood perfusion using Doppler ultrasonography allows the selection of highly receptive recipients and could improve fertility in FTET programs. However, it is necessary to standardize the criteria for evaluation of luteal function in order to have a practical and replicable evaluation by field practitioners. Doppler ultrasonography adoption by veterinarians in cattle reproductive programs will also depend on more studies to improve the hormonal protocols for resynchronization. These features would allow a better diffusion and understanding of the real potential of this biotechnology in ET programs. In addition, the equipment setup and its cost must always be considered to provide accuracy and economic feasibility for this technique.
